# Polymorphisms of *MUC16* (CA125) and *MUC1* (CA15.3) in Relation to Ovarian Cancer Risk and Survival

**DOI:** 10.1371/journal.pone.0088334

**Published:** 2014-02-13

**Authors:** Kristina A. Williams, Kathryn L. Terry, Shelley S. Tworoger, Allison F. Vitonis, Linda J. Titus, Daniel W. Cramer

**Affiliations:** 1 Obstetrics and Gynecology Epidemiology Center, Department of Obstetrics and Gynecology, Brigham and Women’s Hospital, Boston, Massachusetts, United States of America; 2 Harvard Medical School, Boston, Massachusetts, United States of America; 3 Department of Epidemiology, Harvard School of Public Health, Boston, Massachusetts, United States of America; 4 Channing Division of Network Medicine, Department of Medicine, Brigham and Women’s Hospital and Harvard Medical School, Boston, Massachusetts, United States of America; 5 Department of Community & Family Medicine, Department of Pediatrics, Dartmouth-Hitchcock Medical Center Lebanon, New Hampshire, United States of America; University of Nebraska Medical Center, United States of America

## Abstract

**Objective:**

To examine single nucleotide polymorphism (SNPs) in *MUC16* (CA125) and *MUC1* (CA15.3) in relation to ovarian cancer risk and survival.

**Methods:**

We genotyped germline variants of *MUC16* (rs2547065, rs1559168, rs12984471, rs2121133) and *MUC1* (rs2070803, rs4072037, rs1045253) using samples collected from 758 ovarian cancer cases and 788 controls enrolled in the New England Case-Control Study between 2003 and 2008. We calculated age-adjusted odds ratios (OR) and 95% confidence intervals (CIs) for disease risk using unconditional and polytomous logistic regression and hazard ratios (HR) for survival using Cox proportional hazard ratios. In a subset of cases, we compared log-normalized CA125 values by genotype using generalized linear models.

**Results:**

Cases homozygous for the variant allele of *MUC16* SNP, rs12984471, had poorer overall survival (log-rank p = 0.03) and higher CA125 levels, especially cases over age 65 (p = 0.01). For *MUC1* SNP, rs4072037, women homozygous for the G variant had a non-significantly decreased risk for serous invasive types but elevated risk for serous borderline tumors, mucinous borderline and invasive tumors, and endometrioid tumors. Women with the variant allele of *MUC16* SNP, rs2547065, especially those who were homozygous had an elevated risk for ovarian cancer; but this association was not confirmed in an independent dataset.

**Conclusion:**

This targeted screen of seven polymorphisms of *MUC16* and *MUC1* genes failed to identify and confirm effects on ovarian cancer risk overall. However, there may be effects of *MUC16* rs12984471 on survival and *MUC1* rs4072037 on risk for histologic types of ovarian cancer other than invasive serous. Further study is warranted.

## Introduction

The tethered human mucins (MUC) are a family of large, heavily glycosylated transmembrane proteins that have a diverse range of functions [1]. CA125, or MUC16, is the largest glycoprotein of the mucin family, and is normally expressed in the epithelial lining of various tissues, especially that of the female reproductive tract [1]. CA125 is elevated in the serum of about 82% of ovarian cancer patients and is used to predict recurrence [2], [3]. CA15.3, or MUC1, also is expressed in the epithelial lining of various tissues, exhibiting strong expression in the mammary gland and the female reproductive tract during pregnancy and lactation. CA15.3 is over-expressed in a wide variety of cancers, including breast and ovarian [1], [4]. Although these two mucins are best known as tumor markers, evidence suggests that they may play a role in cancer metastasis, tumor growth and survival, inhibition of immune response, and prognosis [1], [5], [6].

Several studies have examined genetic variation in genes involved in glycosylation of CA125 and CA15.3 and ovarian cancer risk, observing overall null associations [7], [8], [9], [10]; however, there are few studies of genetic variation specifically in *MUC16* or *MUC1* and their association with ovarian cancer risk or survival. Therefore, we examined the association between a targeted set of single nucleotide polymorphisms (SNPs) in *MUC16* (rs2547065, rs1559168, rs12914471, rs2121133) and *MUC1* (rs2070803, rs4072037, rs1045253) in relation to ovarian cancer risk and survival.

## Methods

### Ethics Statement

Institutional Review Boards at Brigham and Women’s Hospital, Dana Farber Cancer Institute, and Dartmouth Medical School approved the studies and all study participants signed informed consent.

### Study Population and Design

Data and specimens come from the last enrollment phase of the New England Case Control Study of ovarian cancer from 2003–2008 (NECC). Details regarding case and control enrollment for this study are described elsewhere [11], [12]. Briefly, of 1610 incident cases of ovarian cancer identified through hospital tumor boards and statewide cancer registries between 2003 and 2008, 897 of 1238 eligible agreed to participate. Controls were identified through town books in eastern Massachusetts and drivers’ license lists in New Hampshire. Exclusion criteria for controls included inability to be contacted, history of bilateral oophorectomy, language barriers, or relocation outside of the study area. Of 2522 controls identified, 1673 were eligible and 857 agreed to participate.

After written informed consent, demographic information, reproductive and medical history, and lifestyle factors were assessed by in-person interviews and heparinized blood samples were collected.

### Genotyping

DNA was extracted and genotyping was performed at the Dana-Farber/Harvard Cancer Center (DF/HCC) High Throughput Polymorphism Core, an affiliate of the Partners Healthcare Center for Personalized Genetic Medicine. DNA was extracted from buffy coat samples using QIAmp (Qiagen, Chatworth, CA). Genotyping of *MUC16* (rs2547065, rs1559168, rs12914471, rs2121133) and *MUC1* (rs2070803, rs4072037, rs1045253) was performed using 5′ nuclease assays (Taqman®) on the Applied Biosystems Prism 7900HT Sequence Detection System (Applied Biosystems, Foster City, California). Primers, probes, and conditions for genotyping assays are available upon request. Replicates of 10% of the samples were included for quality control. Laboratory personnel were blinded to case control status and the location of quality controls.

Based on preliminary data, we sought to validate one of the SNPs in an independent dataset. We used samples from 534 cases and 1513 controls from the Nurses’ Health Study cohorts (NHS/NHSII) [13]. The NHS includes 121,700 participants, 32,826 of whom provided blood samples in 1990 and 33,040 who gave buccal cells specimens from 2001–2004. The NHSII includes 116,430 participants, of whom 29,611 provided blood from 1996–1999 and 29,859 provided buccal cells from 2004–2006. Cases were identified after sample collection and before June 1, 2010 (NHS) or June 1, 2009 (NHSII). Demographic information on NHS and NHSII participants have been described previously [13]. Briefly, participants in both cohorts are predominantly white (>96%), but NHS is an older cohort than NHSII which is reflected in participants’ mean age (NHS:65years, NHSII:49years), ever a child birth (95% NHS, 76% NHSII), and ever oral contraceptive use (45% NHS, 81% NHSII).

### Preoperative CA125 Levels

We reviewed all medical records and computerized laboratory reports for cases who received care at Brigham and Women’s Hospital or Massachusetts General Hospital (n = 809) [14]. CA125 values were abstracted for women whose levels had been measured prior to surgery and/or neoadjuvant chemotherapy. We were able to retrieve CA125 values on 353 of the cases genotyped in this study. Data on CA15.3 were not available.

### Statistical Analysis

We used chi-square tests to assess Hardy–Weinberg Equilibrium (HWE) for each SNP among controls. Unconditional logistic regression was used to calculate overall odds ratios (OR) and 95% confidence intervals (95% CI) of ovarian cancer risk adjusted for age (continuous), study center (Massachusetts or New Hampshire), and race (white or non-white). The more common allele for each SNP served as the reference group in the regression models. Co-dominant (heterozygous vs. wild type or homozygous variant vs. wild type), recessive (homozygous variant vs. heterozygous and wild type), and per allele (trend test) models were computed. Multivariate regression models were additionally adjusted for family history of ovarian or early onset breast cancer and a personal history of breast cancer.

Polytomous logistic regression was used to calculate OR (95%CI) for risk of various histological subtypes adjusted for age, study center, and race. Likelihood ratio tests were used to test for heterogeneity across histologic categories (serous borderline, serous invasive [includes high grade transitional cell and mixed serous], mucinous [borderline and invasive], endometrioid or mixed endometrioid/clear cell, clear cell, undifferentiated [includes unspecified and Brenner tumours]) comparing a model that allows the estimate of the association to vary by histologic type to a model that restricts to one estimate of the association for all histologic types.

Cox proportional hazard models (HR) were used to examine the association between each polymorphism and survival, adjusting for study center and race and in a secondary model for stage (I-IV) and histology (serous, non-serous). Co-dominant, recessive, and per allele models were used as described in supplemental methods (Methods S1). The Kaplan–Meier method was used to estimate survival curves and calculate log-rank statistics.

Geometric mean CA125 values by genotype were calculated for each *MUC16* polymorphism. Statistical analyses used general linear regression, adjusted for age, race, and time between CA125 measurement and diagnosis (≤30 days, >30days, missing), using continuous log transformed CA125 levels and a variable that represents increasing variant alleles for each polymorphism (0, 1, 2). CA125 levels can vary during the menstrual cycle, and levels vary between pre and postmenopausal women [15], [16], so we stratified these analyses into three age/menopausal categories (premenopausal, “midlife” postmenopausal (age<65), and “elderly” postmenopausal (age> = 65)). All analyses were performed using SAS v 9.1 (SAS, Cary, North Carolina) and Intercooled Stata 9 (StataCorp LP, College Station, Texas).

## Results

A total of 758 women with ovarian cancer and 788 controls were included in the final analytic sample (Table 1). For both cases and controls, mean age was 54. Our study population consisted primarily of Caucasian women (>95%) and white ethnicity was more common among controls. On average, controls had higher parity, longer duration of oral contraceptive use, and a higher frequency of tubal ligation, endometriosis or painful periods, and a personal history of breast cancer. Family history of ovarian or early onset breast cancer, smoking status, and menopausal status did not differ significantly between cases and controls. Serous invasive (49.7%) was the most frequent histologic subtype among cases followed by endometrioid (17.8%).

**Table 1:** Descriptive characteristics of ovarian cancer cases and controls, New England-based ovarian cancer case-control study 2003–2008.

	Controls		Cases		
Variable	N = 788		N = 758		p^b^
Age, mean (SD)	54.4	(11.8)	54.2	(11.3)	0.77
Study Center					
Massachusetts	670	(85.0)	609	(80.3)	0.01
New Hampshire	118	(15.0)	149	(19.7)	
White Ethnicity, n (%)	774	(98.2)	715	(94.3)	<0.0001
Parous, n (%)	652	(82.7)	522	(68.9)	<0.0001
Mean PregnanciesAmong Parous (SD)	2.6	(1.2)	2.4	(1.2)	0.01
Oral Contraceptive Use, n (%)	541	(68.7)	439	(57.9)	<0.0001
Mean Years Among Users (SD)	6.3	(5.3)	5.1	(5.0)	0.0003
Tubal Ligation, n (%)	176	(22.3)	92	(12.1)	<0.0001
Ever Smoker, n (%)	405	(51.4)	398	(52.5)	0.66
Menopausal Status, n (%)					
Premenopausal	294	(37.3)	278	(36.7)	0.90
Postmenopausal, Age <65	346	(43.9)	333	(43.7)	
Postmenopausal, Age ≥65	148	(18.8)	149	(19.7)	
Endometrioses/Painful Periods,n (%)	279	(35.5)	347	(45.8)	<0.0001
Family History,^a^ n (%)	62	(7.9)	74	(9.8)	0.19
Personal History of BreastCancer, n (%)	35	(4.4)	67	(8.8)	0.0005
Histologic Subtype, n (%)					
Serous Borderline			62	(8.2)	
Serous Invasive			385	(50.8)	
Mucinous			75	(9.9)	
Endometrioid			134	(17.7)	
Clear Cell			50	(6.6)	
Other/Undifferentiated			52	(6.9)	

Cases and controls are frequency matched by age.

a Includes ovarian and early onset (before age 50) breast cancers.

b p value from chi square or t-test.

All seven polymorphisms were in Hardy-Weinberg equilibrium and had genotyping success greater than 95% except for rs2547065 (93%). In general the minor allele frequencies (MAF) we found for our controls were comparable to that of the Caucasian European (CEU) HapMap populations (data not shown). In the NECC study, one of the four *MUC16* polymorphisms was associated with ovarian cancer risk (Table 2). For polymorphism rs2547065, we observed an increase in ovarian cancer risk (per allele OR = 1.26, 95% CI: 1.09–1.47). Risk was most apparent for the homozygous variant genotype when compared to the wild type genotype (OR = 1.68, 95% CI: 1.23–2.29). However, polymorphism rs2547065 was not associated with ovarian cancer risk (per allele: OR = 1.05, 95% CI: 0.91–1.21) in an independent dataset including 534 cases and 1513 controls from the Nurses’ Health Study cohorts (NHS/NHSII). There was no significant heterogeneity by histologic type for any of the *MUC16* polymorphisms.

**Table 2:** Association between mucin polymorphisms and risk of epithelial ovarian cancer, New England-based ovarian cancer case-control study, 2003–2008.

		Controls (N = 788)	All Cases (N = 758)		Serous Invasive Cases (N = 385)		
	MAF	N(%)	N(%)	OR(95% CI)^a^	N (%)	OR (95% CI)^b^	p_het_ c
**MUC16**							
RS12984471	33%						
GG		348 (45.2)	326 (44.7)	1.00 (ref)	158 (43.3)	1.00 (ref)	0.74^d^
CG		338 (43.9)	307 (42.1)	1.01 (0.81, 1.25)	157 (43.0)	1.05 (0.80, 1.37)	
CC		84 (10.9)	97 (13.3)	1.28 (0.92, 1.79)	50 (13.7)	1.36 (0.91, 2.02)	
Per C Allele				1.09 (0.94, 1.27)		1.13 (0.94, 1.36)	0.64^e^
CC vs. GG/GC				1.28 (0.93, 1.75)		1.32 (0.91, 1.93)	0.35^f^
RS1559168	18%						
TT		660 (85.6)	630 (86.3)	1.00 (ref)	322 (87.7)	1.00 (ref)	
AT		107 (13.9)	96 (13.2)	0.92 (0.68, 1.24)	43 (11.7)	–	
AA		4 (0.5)	4 (0.5)	1.06 (0.26, 4.26)	2 (0.5)	–	
Per A Allele				0.94 (0.71, 1.24)		0.84 (0.60, 1.21)	0.36^e^
AA vs. TT/AT				1.07 (0.26, 4.31)		–	
RS2121133	33%						
AA		377 (50.2)	393 (55.0)	1.00 (ref)	193 (53.3)	1.00 (ref)	0.65^d^
AG		318 (42.3)	277 (38.7)	0.84 (0.68, 1.05)	149 (41.2)	0.92 (0.71, 1.20)	
GG		56 (7.5)	45 (6.3)	0.78 (0.51, 1.18)	20 (5.5)	0.69 (0.40, 1.18)	
Per G Allele				0.86 (0.73, 1.02)		0.88 (0.72, 1.08)	0.48^e^
GG vs. AA/AG				0.84 (0.55, 1.26)		0.71 (0.42, 1.21)	0.69^f^
RS2547065	39%						
GG		269 (36.7)	223 (31.9)	1.00 (ref)	113 (32.4)	1.00 (ref)	0.96^d^
GC		359 (49.0)	330 (47.2)	1.11 (0.88, 1.40)	165 (47.3)	1.09 (0.82, 1.46)	
CC		104 (14.2)	146 (20.9)	1.68 (1.23, 2.29)	71 (20.3)	1.61 (1.11, 2.34)	
Per C Allele				1.26 (1.09, 1.47)		1.24 (1.03, 1.48)	0.90^e^
CC vs. GG/GC				1.58 (1.20, 2.09)		1.53 (1.09, 2.13)	0.86^f^
**MUC1**							
RS1045253	30%						
GG		355 (47.7)	322 (44.5)	1.00 (ref)	148 (40.9)	1.00 (ref)	0.63^d^
GA		332 (44.4)	338 (46.7)	1.09 (0.88, 1.35)	180 (49.7)	1.25 (0.96, 1.63)	
AA		58 (7.8)	63 (8.7)	1.13 (0.76, 1.67)	34 (9.4)	1.41 (0.89, 2.24)	
Per A Allele				1.07 (0.91, 1.26)		1.21 (1.00, 1.48)	0.33^e^
AA vs. GG/GA				1.08 (0.74, 1.58)		1.26 (0.82, 1.95)	0.51^f^
RS2070803	48%						
AA		253 (33.1)	228 (31.3)	1.00 (ref)	96 (26.6)	1.00 (ref)	0.30^d^
AG		386 (50.5)	346 (47.5)	0.96 (0.76, 1.21)	186 (51.5)	1.22 (0.91, 1.64)	
GG		125 (16.4)	155 (21.3)	1.27 (0.94, 1.71)	79 (21.9)	1.43 (1.07, 2.22)	
Per G Allele				1.10 (0.95, 1.28)		1.23 (1.03, 1.47)	0.46^e^
GG vs. AA/AG				1.30 (0.99, 1.69)		1.35 (0.99, 1.86)	0.84^f^
RS4072037	47%						
AA		214 (28.3)	226 (31.1)	1.00 (ref)	118 (32.9)	1.00 (ref)	0.06^d^
AG		376 (49.7)	329 (45.3)	0.87 (0.69, 1.11)	174 (48.5)	0.87 (0.65, 1.16)	
GG		167 (22.1)	172 (23.7)	1.06 (0.80, 1.41)	67 (18.7)	0.78 (0.54, 1.11)	
Per G Allele				1.02 (0.88, 1.18)		0.89 (0.74, 1.06)	0.12^e^
GG vs. AA/AG				1.15 (0.90, 1.47)		0.85 (0.62, 1.16)	0.02^f^

a OR (95%CI) modeled with unconditional logistic regression; adjusted for age, study center, and race (white, non-white).

b OR (95%CI) modeled with polytomous logistic regression adjusted for age, study center, and race.

c p-values for heterogeneity (het) are computed with likelihood ratio tests comparing a model that allows the estimate of the association to vary by histologic type (serous borderline, serous invasive, mucinous, endometrioid, clear cell, undifferentiated) to a model that restricts to one estimate of the association for all histologic types.

d p_het_ for co-dominant model.

e p_het_ for per allele model.

f p_het_ for recessive model.

For *MUC1*, none of the polymorphisms we tested were significantly associated with overall ovarian cancer risk in the per allele model. Women carrying two copies of the *MUC1* polymorphism rs2070803 variant had an increased risk of serous invasive cancer of borderline statistical significance (OR = 1.35, 95% CI 0.99–1.86) in the recessive model. Although we observed no significant risk associated with *MUC1* polymorphism rs4072037 overall, significant heterogeneity by histology was observed when evaluating the recessive model for this polymorphism (p-heterogeneity = 0.02). Women homozygous for the G variant of rs4072023 had a non-significantly decreased risk for invasive serous cancers but elevated risks for serous borderline tumors (OR = 1.91, 95% CI 1.08–3.36), mucinous borderline and invasive (OR = 1.68, 95% CI 1.00–2.83), and endometrioid tumors (OR = 1.54, 95% CI 1.01–2.33). Adjusting for family history of ovarian or early onset breast cancer and personal history of breast cancer did not change estimates for any of the *MUC1* or *MUC16* polymorphisms.

**Figure 1:**
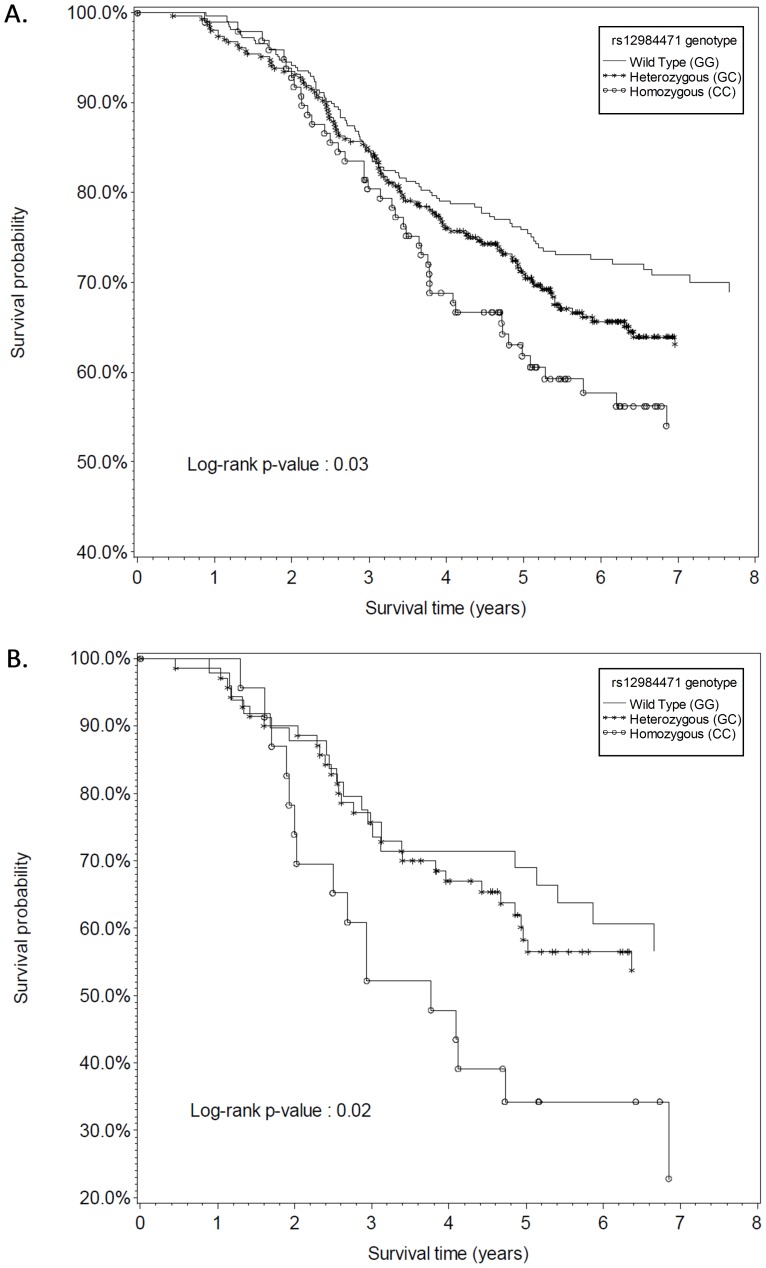
Kaplan–Meier estimates of survival according to rs12984471 genotype among women with epithelial ovarian cancer, New-England based case control study, 2003–2008. **A**. All women **B**. Postmenopausal women age 65 and older.

In examining the effect of mucin polymorphisms and survival, we observed poorer overall survival among women carrying the variant allele of *MUC16* polymorphism rs12984471 (log-rank p = 0.03; Figure 1A) as well as an increased mortality (age-adjusted HR = 1.23 95% CI: 1.02–1.48, Table 3). These associations were strongest in women age 65 and older (log-rank p = 0.02; age-adjusted HR = 1.53 95% CI: 1.07–2.19) (Figure 1B). Mortality was 57% greater for women who were homozygous for the variant allele of rs12984471 (age-adjusted HR = 1.57 95% CI: 1.09–2.28). Adjustment for stage and histology attenuated the association (multivariate HR = 1.32 95% CI: 0.91–1.92); stage was the strongest predictor of survival. No other associations between the remaining mucin polymorphisms and survival were observed.

**Table 3:** Association between mucin polymorphisms and epithelial ovarian cancer survival, New England-based ovarian cancer case-control study, 2003–2008.

	Age-Adjusted	Multivariate	Age-Adjusted	Multivariate
	HR (95% CI)^a^	HR (95% CI)^b^	HR (95% CI)^a^	HR (95% CI)^b^
**MUC16**				
RS12984471				
GG	1.00 (ref)	1.00 (ref)	1.00 (ref)	1.00 (ref)
CG	1.13 (0.85, 1.50)	1.13 (0.85, 1.51)	1.16 (0.85, 1.58)	1.15 (0.85, 1.57)
CC	1.57 (1.09, 2.28)	1.32 (0.91, 1.92)	1.17 (0.75, 1.81)	1.11 (0.72, 1.73)
Per C Allele	1.23 (1.02, 1.48)	1.15 (0.96, 1.37)	1.10 (0.90, 1.35)	1.08 (0.88, 1.32)
CC vs. GG/GC	1.48 (1.06, 2.07)	1.25 (0.89, 1.75)	1.08 (0.72, 1.63)	1.04 (0.69, 1.56)
RS1559168				
TT	1.00 (ref)	1.00 (ref)	1.00 (ref)	1.00 (ref)
AT	1.00 (0.68, 1.47)	1.13 (0.77, 1.67)	1.15 (0.75, 1.76)	1.16 (0.76, 1.77)
AA	–	–	–	–
Per A Allele	0.89 (0.61, 1.28)	1.02 (0.70, 1.47)	1.00 (0.67, 1.48)	1.04 (0.69, 1.54)
AA vs. TT/AT	–	–	–	–
RS2121133				
AA	1.00 (ref)	1.00 (ref)	1.00 (ref)	1.00 (ref)
AG	0.86 (0.65, 1.12)	0.86 (0.65, 1.13)	0.92 (0.68, 1.24)	0.91 (0.68, 1.23)
GG	0.94 (0.54, 1.64)	0.95 (0.55, 1.65)	1.21 (0.66, 2.21)	1.11 (0.61, 2.02)
Per G Allele	0.91 (0.73, 1.13)	0.91 (0.73, 1.13)	1.00 (0.78, 1.27)	0.98 (0.77, 1.24)
GG vs. AA/AG	1.01 (0.59, 1.73)	1.01 (0.59, 1.74)	1.26 (0.70, 2.26)	1.16 (0.64, 2.08)
RS2547065				
GG	1.00 (ref)	1.00 (ref)	1.00 (ref)	1.00 (ref)
GC	1.02 (0.76, 1.39)	0.97 (0.71, 1.31)	1.03 (0.74, 1.43)	0.99 (0.71, 1.38)
CC	1.04 (0.71, 1.51)	0.93 (0.64, 1.36)	0.76 (0.49, 1.18)	0.74 (0.47, 1.15)
Per C Allele	1.02 (0.85, 1.23)	0.97 (0.80, 1.16)	0.90 (0.73, 1.10)	0.88 (0.71, 1.08)
CC vs. GG/GC	1.02 (0.74, 1.42)	0.95 (0.69, 1.32)	0.75 (0.50, 1.11)	0.74 (0.50, 1.10)
**MUC1**				
RS1045253				
GG	1.00 (ref)	1.00 (ref)	1.00 (ref)	1.00 (ref)
GA	1.25 (0.94, 1.65)	1.12 (0.85, 1.47)	1.03 (0.75, 1.41)	1.05 (0.77, 1.42)
AA	0.99 (0.60, 1.65)	0.89 (0.54, 1.47)	0.78 (0.45, 1.35)	0.81 (0.48, 1.37)
Per A Allele	1.07 (0.88, 1.30)	1.01 (0.82, 1.24)	0.95 (0.76, 1.19)	0.95 (0.76, 1.19)
AA vs. GG/GA	0.89 (0.55, 1.44)	0.84 (0.53, 1.35)	0.77 (0.46, 1.29)	0.79 (0.48, 1.30)
RS2070803				
AA	1.00 (ref)	1.00 (ref)	1.00 (ref)	1.00 (ref)
AG	0.90 (0.67, 1.21)	0.83 (0.62, 1.12)	0.80 (0.58, 1.12)	0.87 (0.63, 1.22)
GG	1.06 (0.74, 1.52)	1.02 (0.71, 1.47)	1.02 (0.69, 1.52)	1.05 (0.70, 1.57)
Per G Allele	1.01 (0.84, 1.22)	0.99 (0.82, 1.20)	0.99 (0.81, 1.23)	1.01 (0.82, 1.25)
GG vs. AA/AG	1.12 (0.82, 1.54)	1.15 (0.84, 1.57)	1.18 (0.84, 1.66)	1.15 (0.81, 1.61)
RS4072037				
AA	1.00 (ref)	1.00 (ref)	1.00 (ref)	1.00 (ref)
AG	1.03 (0.76, 1.38)	1.02 (0.76, 1.38)	1.10 (0.80, 1.52)	1.16 (0.85, 1.60)
GG	0.84 (0.58, 1.21)	0.89 (0.61, 1.28)	0.85 (0.56, 1.30)	0.82 (0.53, 1.25)
Per G Allele	0.93 (0.78, 1.11)	0.95 (0.79, 1.14)	0.95 (0.78, 1.16)	0.94 (0.77, 1.14)
GG vs. AA/AG	0.83 (0.60, 1.14)	0.87 (0.64, 1.20)	0.80 (0.55, 1.17)	0.74 (0.51, 1.09)

Modeled with Cox proportional hazard ratios; “–” frequencies for this SNP were too low to compute co-dominant/recessive models.

a Adjusted for age, study center, and race.

b Adjusted for age, study center, race, stage (I-IV) and histologic subtype (non-serous, serous).

Finally, we evaluated the association between *MUC16* polymorphisms and serum levels of CA125 measured preoperatively (Table 4). Among all cases, we observed no linear associations between *MUC16* polymorphisms and CA125 levels; however, rs2121133 had the highest CA125 levels for heterozygotes (319.9) and lowest for homozygous variants (110.0) (p = 0.03). Among elderly postmenopausal women, increasing variant alleles of polymorphism rs12984471 were significantly associated with increasing levels of CA125 (p = 0.02).

**Table 4:** Geometric mean levels of preoperative serum CA125 levels among women with ovarian cancer by MUC16 gene polymorphisms, New England-based ovarian case-control study, 2003–2008.

	All Women			Premenopausal			Postmenopausal <65			Postmenopausal ≥65		
	N	GM	p^a^	N	GM	p^a^	N	GM	p^a^	N	GM	p^a^
RS12984471												
GG	145	193.5	0.15	58	139.9	0.94	63	265.8	0.64	24	184.0	0.02
CG	145	254.6		41	166.2		65	290.7		39	319.9	
CC	49	398.4		14	163.2		22	423.6		13	939.4	
RS1559168												
TT	303	242.1	0.68	98	161.9	0.98	133	277.5	0.40	72	325.5	0.89
AT	34	302.4		12	124.5		15	527.2		7	420.8	
AA	1	102.9		0	–		1	102.9		0	–	
RS2121133												
AA	201	226.7	0.03	62	141.9	0.06	89	288.4	0.90	50	263.8	0.07
AG	121	319.9		45	224.5		52	313.4		24	650.1	
GG	17	110.0		5	25.7		7	245.5		5	153.1	
RS2547065												
GG	98	194.2	0.16	39	192.5	0.10	41	166.5	0.06	18	281.6	0.74
GC	166	290.3		50	147.5		71	393.5		45	381.2	
CC	67	186.9		20	88.9		32	232.6		15	315.4	

Abbreviations: GM = Geometric mean.

a modeled with general linear regression; adjusted for age, study center, race and time between CA125 to diagnosis (≤30days, >30days, missing).

## Discussion

The *MUC1* gene is located on 1q21–22, which is a region frequently altered in both neoplastic and non-neoplastic disorders. *MUC1* gene amplification due to increased gene copy number has been observed in ovarian, breast, papillary thyroid, and gastric cancers [17], [18], [19], [20]. Neoplastic mammary cells have been shown to have a high frequency of altered DNA within the variable nucleotide repeat region (VNTR) of MUC1- the largest region of the this protein and the site of O-glycosylation [1], [21], [22]. MUC1 has been shown to be essential for ovarian cancer tumorigenesis in mouse models and is over expressed in approximately 90–100% of serous carcinomas [23], [24], [25]. The three *MUC1* SNPs we studied were selected based on previous publications that studied associations between *MUC1* polymorphisms and gastrointestinal cancers [26], [27]. Polymorphism rs4072037 has been correlated with serum MUC1 levels and is known to play a role in alternative splicing [28], [29]. Polymorphism rs2070803 is located upstream of the *MUC1* gene in a large LD block, and polymorphism rs1045253 was previously identified as a tagSNP representative of the *MUC1* region [30], [31], [32]. None of these three polymorphisms affected risk or survival for ovarian cancer overall. Women who were homozygous for the variant G allele of rs2070803 had a 35% elevation in risk for invasive serous ovarian cancer. Although our finding was of borderline statistical significance, a Japanese study found risk for “diffuse” type gastric cancer to be increased with possession of the G allele [31]. In tests we did for heterogeneity by histologic type of ovarian cancer, only rs4072037 varied with increased risks for types other than invasive serous including especially serous borderline, mucinous, and endometrioid. In general, all histologic types of epithelial ovarian tumors, both benign and malignant, express MUC1 on the cell surface by immunohistochemistry [33]. However a recent study using gene expression proposed that MUC1 expression is low in one subtype of invasive serous tumors [34].

The *MUC16* gene is located at 19p13, which is altered in a variety of cancers, especially ovarian. In ovarian carcinoma, 19p13 has been identified as the chromosome band most frequently involved in structural rearrangement [35], [36]. This region has also been shown to be highly amplified in high grade serous carcinoma [37]. Polymorphisms rs12984471, rs1559168, rs2121133 are tagSNPs and were selected for our study because they are representative of various regions of the *MUC16* gene. The minor alleles of rs12984471 and rs1559168 introduce missense mutations while rs2121133 is within an intron [38]. Polymorphism rs2547065 was selected because it was previously studied in relation to epithelial ovarian cancer [39] and introduces a missense mutation that could potentially contribute to a functional modification of the gene product. Among the *MUC16* polymorphisms studied, we observed associations between rs2547065 and ovarian cancer risk and between rs12984471 and survival.

In a small study that examined two *MUC16* variants including rs2547065, Bouanene et al. observed that the CC genotype was more frequent in cases (49%) than in controls (34%), similar in direction to what we observed but not significant in their study which included only 41 cases and 76 controls [39]. Despite the fact that homozygous variant genotype (CC) of polymorphism rs2547065 was associated with ovarian cancer risk overall and invasive serous ovarian cancer in the NECC data, we were unable to validate this finding in independent data from the Nurses’ Health Study. With positive results from our study, supportive results from the only published study related to this SNP, but null results from the NHS, validation will be necessary and is planned within the Ovarian Cancer Association Consortium.

*MUC16* polymorphism rs12984471 was the only SNP associated with survival. The variant allele, which conferred poorer survival among all cases, also was correlated with high CA125 serum levels in postmenopausal women, especially those over the age of 65. The possibility that the association between rs12984471 and survival may have a biologic basis is suggested by possible functional consequences on MUC16 protein. Polymorphism rs12984471 is located in the largely uncharacterized extracellular subunit where the C variant is responsible for a conservative missense mutation changing a glutamate to an aspartate [38], [40]. Conservative amino acid changes are theorized to confer 40% likelihood of gene function modification compared to a silent mutation [41]. Thus, it is plausible that the missense mutations caused by this polymorphism could influence the function of the *MUC16* gene product as suggested by its effects on CA125 levels in cases.

In conclusion, our study examined four SNPs in *MUC16* (CA125) and three SNPs in *MUC1* (CA15.3) in relation to ovarian cancer risk and survival in the New England Case-Control study. One of four *MUC16* SNPs, rs2547065, was associated with increased risk for ovarian cancer. A different *MUC16* SNP, rs12984471, was associated with survival and also correlated with serum levels of CA125. Of the three *MUC1* SNPs studied, we found one, rs4072037, which displayed significant heterogeneity by histologic type. We had the opportunity to examine one of these findings, rs2547065 with increased risk, in data from the Nurse’s Health Study. The finding was not validated raising the issue of chance in explaining our findings. However, the association of rs12984471 in *MUC16* with survival has some biologic support in that it is also correlated with CA125 levels. Since our study population is composed of primarily Caucasian women, we were not able to generalize our results to other ethnicities. Our study should not be considered definitive because we targeted SNPs as opposed to conducting a comprehensive gene or genome wide investigation. Evaluation of a broader set of tagging SNPs is planned in the Ovarian Cancer Association Consortium.

## Supporting Information

Methods S1 Supplementary methods on age and histologic type classification, statistical models, and SNP selection and genotyping.
(DOCX)
