# Supplementary material for: Polymorphisms of MUC16 (CA125) and MUC1 (CA15.3) in Relation to Ovarian Cancer Risk and Survival
Source: PLoS One. 2014 Feb 13;9(2):e88334. doi: 10.1371/journal.pone.0088334 (PMC3923771; doi:10.1371/journal.pone.0088334)
Supplement: Methods S1 — Supplementary methods on age and histologic type classification, statistical models, and SNP selection and genotyping. (DOCX) [file pone.0088334.s001.docx]

**Supplemental Methods**

**Age and histological categories**

Women were classified as premenopausal if their periods were still occurring spontaneously or they were pregnant or breastfeeding or using oral contraceptives (OCs) or progesterone. Women who had hysterectomy (prior to the ovarian cancer treatment for cases) were considered premenopausal if younger than 50. Women were considered postmenopausal if their periods had stopped at least one year prior to the date of enrolment or were occurring because of hormone replacement for menopause, and women who had hysterectomy were classified as postmenopausal if over the age of 50. Postmenopausal women were further divided into two groups based on age; a “midlife” group (age<65) and an “elderly” group (age>65) [1].

Histological subtype was abstracted from pathology reports which were reviewed for consistency by a single pathologist. Specific subgroups included serous borderline, serous invasive, mucinous (borderline and invasive), endometrioid, and clear cell tumors. Invasive and borderline classifications were based on tumor grades and all undifferentiated counted as malignant. Mixed tumors described as “predominantly” one type or containing “focal” area of another were coded to the predominant type. Transitional cell tumors or mixed serous and transitional tumors were included with serous. Mixed endometrioid and clear cell tumors were included with endometrioid. Other mixed epithelial tumors, malignant Brenner tumors, unspecified epithelial and undifferentiated tumors were grouped as “other/undifferentiated”.

**Statistical Models for estimating SNP associations**

In the co-dominant model, we compared women carrying one copy of the variant allele (heterozygous) or women carrying two copies of the variant allele (homozygous variant) to women carrying two common alleles (wild type) (heterozygous vs. wild type or homozygous vs. wild type). In the recessive model, we compared women carrying two copies of the minor allele to women carrying one or two common alleles (homozygous variant vs. heterozygous or wild type). In the per minor allele model, trend tests were calculated using a variable representing the number of variant alleles (0, 1, or 2).

**SNP selection**

We studied four tagSNPs of the *MUC16* gene- rs2547065 (G/C), rs1559168 (T/A), rs12984471 (G/C), and rs2121133 (A/G)- and three SNPs associated with the *MUC1* gene- rs1045253 (G/A), rs2070803 (A/G), rs4072037 (A/G). *MUC16* polymorphisms rs1559168(T/A), rs12984471 (G/C), and rs2121133 (A/G), were identified through HapMap (<http://www.hapmap.org>) using Tagger for SNP selection [2]. Polymorphism rs2547065 (G/C) was identified through literature review [3]. *MUC1* polymorphism rs4072037 (A/G) is a functional SNP [4] and rs1045253 (G/A) is a tagSNP of the *MUC1* gene; the *MUC1* gene is found in the LD block of polymorphism rs2070803 (A/G). All *MUC1* SNPs were identified through literature review [5-7]. We used dbSNP (<http://www.ncbi.nlm.nih.gov/projects/SNP/>) to compare frequency and variant data on all SNPs.

References

1. McKenna RJ, Sr. (1994) Clinical aspects of cancer in the elderly. Treatment decisions, treatment choices, and follow-up. Cancer 74: 2107-2117.

2. de Bakker PI, Yelensky R, Pe'er I, Gabriel SB, Daly MJ, et al. (2005) Efficiency and power in genetic association studies. Nat Genet 37: 1217-1223.

3. Bouanene H, Hadj Kacem H, Ben Fatma L, Ben Limem H, Ben Ahmed S, et al. (2011) Polymorphisms in the MUC16 gene: potential implication in epithelial ovarian cancer. Pathol Oncol Res 17: 295-299.

4. Ng W, Loh AX, Teixeira AS, Pereira SP, Swallow DM (2008) Genetic regulation of MUC1 alternative splicing in human tissues. Br J Cancer 99: 978-985.

5. Saeki N, Saito A, Choi IJ, Matsuo K, Ohnami S, et al. (2011) A functional single nucleotide polymorphism in mucin 1, at chromosome 1q22, determines susceptibility to diffuse-type gastric cancer. Gastroenterology 140: 892-902.

6. Marin F, Bonet C, Munoz X, Garcia N, Pardo ML, et al. (2012) Genetic variation in MUC1, MUC2 and MUC6 genes and evolution of gastric cancer precursor lesions in a long-term follow-up in a high-risk area in Spain. Carcinogenesis 33: 1072-1080.

7. Shi Y, Hu Z, Wu C, Dai J, Li H, et al. (2011) A genome-wide association study identifies new susceptibility loci for non-cardia gastric cancer at 3q13.31 and 5p13.1. Nat Genet 43: 1215-1218.
